# Phytochemical Profile, Antioxidant Activity, and Cytotoxicity Assessment of *Tagetes erecta* L. Flowers

**DOI:** 10.3390/molecules26051201

**Published:** 2021-02-24

**Authors:** Ana Flavia Burlec, Łukasz Pecio, Solomiia Kozachok, Cornelia Mircea, Andreia Corciovă, Liliana Vereștiuc, Oana Cioancă, Wiesław Oleszek, Monica Hăncianu

**Affiliations:** 1 Department of Drug Analysis, Faculty of Pharmacy, “Grigore T. Popa” University of Medicine and Pharmacy, 16 University Street, 700115 Iasi, Romania; flavia_burlec@hotmail.com; 2 Department of Biochemistry and Crop Quality, Institute of Soil Science and Plant Cultivation—State Research Institute, Czartoryskich 8, 24-100 Puławy, Poland; lpecio@iung.pulawy.pl (Ł.P.); skozachok@iung.pulawy.pl (S.K.); wieslaw.oleszek@iung.pulawy.pl (W.O.); 3 Department of Pharmaceutical Biochemistry and Clinical Laboratory, Faculty of Pharmacy, “Grigore T. Popa” University of Medicine and Pharmacy, 16 University Street, 700115 Iasi, Romania; 4 Department of Biomedical Sciences, Faculty of Medical Bioengineering, “Grigore T. Popa” University of Medicine and Pharmacy, 16 University Street, 700115 Iasi, Romania; liliana.verestiuc@umfiasi.ro; 5 Department of Pharmacognosy, Faculty of Pharmacy, “Grigore T. Popa” University of Medicine and Pharmacy, 16 University Street, 700115 Iasi, Romania; oana.cioanca@gmail.com (O.C.); mhancianu@yahoo.com (M.H.)

**Keywords:** *Tagetes erecta* L., Asteraceae, HR-QTOF/MS, polyphenols, quercetagitrin, lipoxygenase

## Abstract

*Tagetes erecta* L. is a popular ornamental plant of the Asteraceae family, which is widely cultivated not only for its decorative use, but also for the extraction of lutein. Besides carotenoid representatives, which have been extensively studied, other important classes of secondary metabolites present in the plant, such as polyphenols, could exhibit important biological activities. The phytochemical analysis of a methanolic extract obtained from *T. erecta* inflorescences was achieved using liquid chromatography–mass spectrometry (LC-MS) techniques. The extract was further subjected to a multistep purification process, which allowed the separation of different fractions. The total extract and its fractions contain several polyphenolic compounds, such as hydroxybenzoic and hydroxycinnamic acid derivatives, flavonols (especially quercetagetin glycosides), and several aglycons (e.g., quercetin, patuletin). One of the fractions, containing mostly quercetagitrin, was subjected to two different antioxidant assays (metal chelating activity and lipoxygenase inhibition) and to in vitro cytotoxicity assessment. Generally, the biological assays showed promising results for the investigated fraction compared to the initial extract. Given the encouraging outcome of the in vitro assays, further purification and structural analysis of compounds from *T. erecta* extracts, as well as further in vivo investigations are justified.

## 1. Introduction

*Tagetes erecta* L. is one of the most popular plants of the Asteraceae family, being native to Central America (especially Guatemala and Mexico). It was introduced to Europe in the 16th century, having been brought from Mexico [1]. Nowadays, it is cultivated all around the world as an ornamental plant, as well as for its high content in carotenoids, which have various uses in the food and pharmaceutical sectors [2]. The species has many synonyms, and can be found under different common names: American Marigold, African Marigold, Aztec Marigold, or Mexican Marigold. Its Spanish and Portuguese common names, “flor de muerto” and “cravo de defuntos”, respectively, refer to the common practice in Central America of using an aqueous extract of the plant for washing bodies after death, given its pleasant and intense smell, as well as to the fact that in that area the plant can be found quite often in cemeteries [3].

Plants from the Tagetes genus have been widely cultivated for their ornamental role, given the fact that such species bloom throughout the summer until the first frost in autumn months [4]. They are usually used for landscaping, but are also grown in plant pots or used as cut flowers. *T. erecta* has fast growth and well-developed roots, being able to grow even on grounds with lower amounts of plant nutrients [5]. The species is also considered quite valuable given its phytoremediation properties, since it exhibits the capacity to accumulate heavy metals like chromium, copper, cadmium, and lead [6,7,8].

*T. erecta* is an annual herbaceous plant with yellow or orange flowers grouped in solitary inflorescences. Given its high content in lutein, which has various applications, the plant has been mostly investigated for its carotenoidic representatives [9]. It is estimated that over 7000 ha are used worldwide for the cultivation of this species, each plant being able to provide around 330 mg xanthophylls. The extracted lutein is used for commercial purposes as a food additive for chickens to color egg yolks [10]. Moreover, it is used for the manufacture of nutritional supplements meant to prevent the loss of visual acuity caused by age-related macular degeneration or by other eye diseases [11]. The plant’s volatile oil has also been studied and is used in the food and cosmetic industries. However, other classes of compounds found in the plant can present important biological actions, such as antioxidant, antiproliferative, and antidiabetic. Such assumptions are justified by the long use of the plant as part of the traditional medicine of different countries from Central and South America. In this context, various extracts of the plant have been administered externally or internally for disorders ranging from skin wounds to inflammatory diseases. Therefore, we decided to investigate the species’ potential use as a resource of polyphenolic acids and flavonoids with biological actions, by conducting a complex profiling of a methanolic extract of *T. erecta* flowers, followed by comparative biological testing of the extract and one of its representative fractions, which, to the best of our knowledge, has been done for the first time through the chosen methods.

## 2. Results

### 2.1. Identification of Compounds Found in the T. erecta Extract

**Figure 1:**
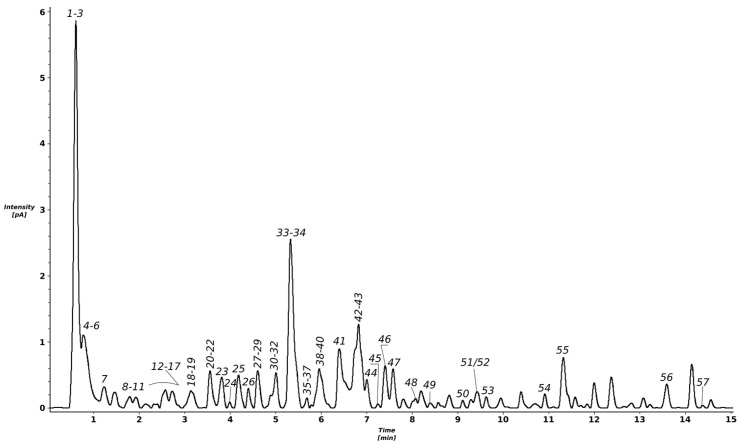
UHPLC–charged aerosol detector (CAD) profile of the *T. erecta* total methanolic extract.

The chromatographic analysis of the methanolic extract obtained from *T. erecta* flowers indicates the presence of several peaks, out of which most were identified as polyphenolic derivatives (Figure 1 and Table 1; peak numbers assigned considering the retention time). More than 50 compounds were tentatively identified using accurate mass measurements, fragmentation patterns, retention times, UV–Vis (ultraviolet–visible) spectra, and data from the literature.

A multistep purification process involving solid-phase extraction and gel permeation column chromatography led to the isolation of four fractions (Fr 1–4). The first separated fraction contained as main components syringic acid (*m/z* = 197.1) and some of its derivatives, such as syringic acid-(hydroxytrimethoxybenzoic acid)-hexoside with *m/z* = 569.2, while fraction 2 contained mostly other syringic acid derivatives, such as di-syringic acid hexoside (*m/z* = 539.1). Fraction 3 was generally comprised of digalloyl-hexoside (*m/z* = 483.1) and ellagic acid hexoside (*m/z* = 463.1), as well as a quercetagetin hexoside (*m/z* = 479.1), and quercetin, while over 85% of the last fraction (Fr 4) contained a quercetagetin hexoside, later confirmed as quercetagitrin through nuclear magnetic resonance (NMR) spectroscopy, with *m/z* = 479.1. The identification of quercetagitrin is detailed in the next section. Compounds identified in the total methanolic extract are listed in Table 1 and can be found in some of the separated fractions.

### 2.2. Identification of Quercetagitrin

The main phenolic component, numbered **34** (retention time around 5.5 min in the CAD chromatogram), which was visible in the UV chromatogram of the *T. erecta* methanolic extract, is a compound possessing two distinctive UV absorption maxima at 260 nm (Band II) and 355 nm (Band I), and its negative-ion HR-QTOF-ESI-MS spectrum showed deprotonated molecules at *m/z* = 479.0836. On this basis, its molecular formula was determined as C_21_H_20_O_13_. Its MS/MS spectra gave fragment ions at *m/z* = 317.0304 [Aglycone−H]^−^ (−162 u = C_6_H_10_O_5_ = hexose), thus suggesting glycosylation of a 6-hydroxyquercetin type of flavonol. The analysis of the ^13^C–NMR spectrum of compound **34** showed 21 signals, sorted by the distortionless enhancement by polarization transfer with retention of quaternaries (DEPTQ) and heteronuclear single quantum coherence (HSQC) experiments into 1 CH_2_, 9 CH, and 11 quaternary carbons. The aromatic region ^1^H and 2D COSY (correlation spectroscopy) NMR spectra exhibited an ABX-type spin system (δ 7.70 (d, *J* = 2.2) = H-2′, 6.89 (d, *J* = 8.5) = H-5′ and 7.53 (dd, *J* = 8.5, 2.2) = H-6′) of ring B and a proton resonance at δ 6.92 (s) = H-8, suggesting a penta-substituted ring A of the flavonol moiety (Table 2). Additionally, a doublet resonating at δ 5.01 with *J* = 7.3 Hz, attributable to a saccharide moiety, was correlating with a series of protons in the 1D TOCSY (total correlation spectroscopy) with large vicinal coupling constants (*J* ≈ 9 Hz), suggesting the presence of a glucose moiety with a β-oriented anomeric proton, as was evidenced by the measurement of direct ^1^H–^13^C ^1^*J* coupling constant (*J* ≈ 160 Hz) [32] in the F2-coupled HSQC experiment [33]. The latter value suggested that the β-Glcp was not attached to the C-3 (δ 135.6) of the aglycon [34], and this was confirmed by the long-range correlation visible in the heteronuclear multiple bond correlation (HMBC) spectrum between H-1_Glcp_ and C-7 (δ 151.5). Overall, the data and the literature reports confirmed that this compound was a quercetagetin-7-*O*-β-glucopyranoside, which is called quercetagitrin. ^1^H- and ^13^C-NMR spectra of this compound are available in the Supplementary Materials.

### 2.3. Antioxidant Activity Assays

The antioxidant testing of the total methanolic extract of *T. erecta* flowers and the investigated fraction (Fr 4) containing over 85% quercetagitrin was carried out comparatively using two renowned methods: 15-lipoxygenase (15-LOX) inhibition assay and metal chelation activity test. The samples’ capacities to chelate iron ions, as well as to inhibit lipoxygenase, were expressed using EC_50_ and IC_50_ values (Table 3). The obtained results were also presented in comparison to those of the positive control (quercetin), in order to evaluate their effectiveness.

Fr 4 presented the most promising lipoxygenase inhibition activity (16.49 ± 0.19 μg/mL final solution), the obtained value being even lower than that of quercetin, which was used as a positive control.

However, regarding the iron-chelating activity, the total extract presented a better activity overall (0.390 ± 0.001 mg/mL final solution), comparable to that of quercetin, while Fr 4 showed lower antioxidant activity through this mechanism (0.529 ± 0.001 mg/mL final solution).

### 2.4. Cytotoxicity Testing

**Figure 2:**
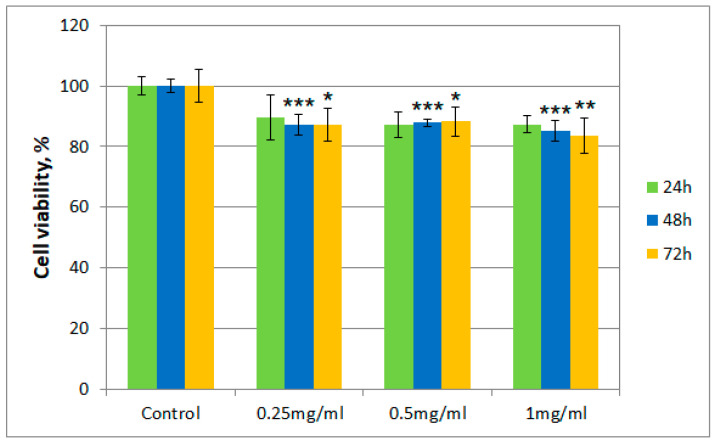
Cell viability using the MTT assay for *T. erecta* total extract at different concentrations. Values are expressed as the mean ± standard error of the mean from three independent experiments (*n* = 3). * *p* < 0.01, ** *p* < 0.001, *** *p* < 0.0001 versus control (analyzed by means of two-way ANOVA).

**Figure 3:**
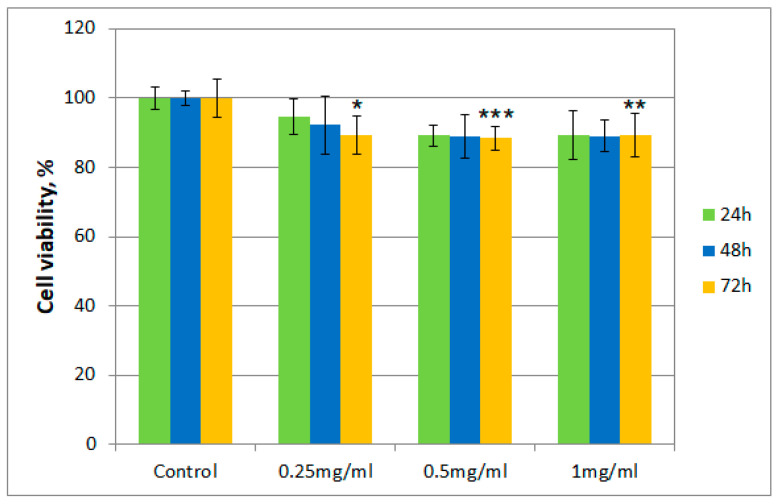
Cell viability using the MTT assay for Fr 4 at different concentrations. Values are expressed as the mean ± standard error of the mean from three independent experiments (*n* = 3). * *p* < 0.01, ** *p* < 0.001, *** *p* < 0.0001 versus control (analyzed by means of two-way ANOVA).

After incubation of albino rabbit dermal fibroblasts with the tested samples (*T. erecta* total extract and Fr 4), the presence of viable cells was evaluated after 24, 48, and 72 h, respectively, using the thiazolyl blue tetrazolium bromide (MTT) assay. Figure 2 and Figure 3 describe the results regarding cell viability of the culture exposed to different concentrations of the samples (0.25 mg/mL, 0.5 mg/mL, and 1 mg/mL, respectively) compared to the control.

Cell viability for both samples was over 80% in the 0.25–0.50 mg/mL concentration range, even after 72 h. However, for cells exposed to a concentration of 1 mg/mL of the *T. erecta* total extract, a slight decrease was noticeable after 48 and 72 h. On the other hand, for Fr 4, such a decrease was not observed with the increase of concentration, with the value for the viability of cells remaining around 90%.

## 3. Discussion

During phytochemical analyses of the total extract, over 50 compounds were tentatively identified, which pertain to different classes of natural metabolites, such as amino acids (phenylalanine, tryptophan), gallic acid, quinic acid, syringic acid and ellagic acid derivatives, and mono- and diacyl chlorogenic acids, as well as flavonoids, especially the glycosides of quercetagetin, quercetin, kaempferol, and patuletin, but also their aglycons (quercetin, kaempferol, patuletin, isorhamnetin, and axillarin). Some of these compounds have been previously reported in the literature for *T. erecta,* particularly quercetagetin and some of its derivatives, syringic acid [36], quercetin, kaempferol [37], ellagic acid [38], and gallic acid and its derivatives [39]. However, to the best of our knowledge, this is the first complete profiling of a methanolic extract of *T. erecta* flowers, followed by a comparative biological analysis of the total extract and one of its fractions through the chosen methods.

Ferrous ions are indirectly involved in the emergence of oxidative stress, taking into account that they partake in biochemical reactions that generate hydroxyl ions. These ions possess a special chemical reactivity and can participate in the initiation of oxidation reactions, especially of unsaturated compounds, thereby modifying the structure of cell membranes. By chelating ferrous ions, the number of available ions for the Fenton reaction decreases, which demonstrates the antioxidant potential that chelating agents have [40]. The aforementioned mechanism represents one of the possibilities through which substances with hydroxyl, carbonyl, or amino groups can act as antioxidants [41,42].

Flavonoids are compounds that have a remarkable capacity for chelating pro-oxidant metal ions like Cu^2+^ or Fe^2+^, which contributes to their antioxidant activity. A large number of representatives can form stable complexes with metals through their hydroxyl groups or even carbonyl groups, when present. For example, quercetin can form stable combinations due to three potential chelation sites: α-hydroxy-carbonyl groups, β-hydroxy-carbonyls, or catechol [40,43]. The obtained results indicate better chelating activity for the methanolic extract, compared to the investigated fraction (Fr 4). Implicitly, the total extract obtained from *T. erecta* presented the lowest EC_50_ value, even lower than that of quercetin, which was used as the positive control. This can be justified by the fact that the total extract generally contains far more polyphenolic compounds than selective fractions, which is equivalent to a larger number of hydroxyl groups that can form complexes with metal ions. Moreover, the species is well-known for its high carotenoidic content, such constituents being able to contribute to the demonstrated antioxidant effect through this mechanism [44].

Lipoxygenases are enzymes involved in the progression of inflammatory processes, and they act as mediators of bronchoconstriction and sensitization reactions. Therefore, LOX inhibitors are potential modulators of such phenomena, and could be of use in the control of inflammation, some gastrointestinal disorders, or adverse cardiovascular reactions. It is suggested that many LOX inhibitors are antioxidant substances and act as free radical scavengers. The interaction between natural polyphenols and the enzyme is of high importance, given the fact that it is considered the potential target through which the biological action of such compounds manifests itself. Consequently, LOX inhibitors have been studied as potential therapeutic agents for the treatment of inflammatory and allergic disorders [45].

The activity of 15-LOX can also be partially or entirely inhibited by compounds that have the capacity of blocking the reversible oxidation of Fe^2+^ to Fe^3+^, and consequently, the enzyme will no longer be able to catalyze the transformation of the substrate through oxidation–reduction reactions. Compounds that act upon the enzyme through this mechanism have a reduction potential, releasing electrons and protons [46,47]. Polyphenols and flavonoids found in plant extracts and fractions have inhibitory activity over the enzyme through the aforementioned mechanism, which is a well-known fact in the scientific literature [45].

It can be easily observed that various methods of separation applied to the total extract prove to be useful for obtaining fractions with enhanced antioxidant capacity. This general remark can be explained by the loss of certain compounds that interfere with the inhibitory action over the enzyme, after separation. In this case, Fr 4, which contains as major constituent quercetagitrin, presented enhanced antioxidant properties through this mechanism.

Moreover, regarding cytotoxicity testing, for the total extract obtained from *T. erecta* flowers, a slight tendency of reduction in cell viability was observed with the concentration and exposure time increase from 24 to 72 h, which could indicate the existence of a cytotoxic potential at higher doses. However, the data presented for Fr 4 suggest that the investigated fraction is not cytotoxic in the 0.25–1.00 mg/mL concentration range, with cell viability maintaining a value of at least 80% of that obtained for the control. This finding implies that, in a reasonable concentration range, the investigated fraction does not possess significant cytotoxicity in the case of albino rabbit dermal fibroblasts.

Therefore, the analyses performed in this study reflect the advantage and importance of separations carried out on *T. erecta* flower extracts, in order to identify new compounds with therapeutic potential, given their improved antioxidant and anti-inflammatory capacities compared to the crude extract, as well as their lowered cytotoxicity.

## 4. Materials and Methods

### 4.1. Chemicals and Reagents

Acetonitrile (LC-MS grade) and methanol (HPLC grade) were acquired from Merck (Darmstadt, Germany), while formic acid (MS-grade) was purchased from Sigma Aldrich (Steinheim, Germany). Ultrapure water was obtained using a Milli-Q Simplicity 185 water purification system (Millipore, Milford, MA, United States). Regarding antioxidant assays, lipoxidase from Glycine max (type I-B), linoleic acid, quercetin, and DMSO were purchased from Sigma Aldrich (Steinheim, Germany), while acetate buffer 0.1 M (pH 5.25) was prepared by mixing sodium acetate 0.1 M solution with acetic acid (Sigma Aldrich) until the appropriate value of pH was reached. Borate buffer (pH 9) was obtained similarly by mixing boric acid (Sigma Aldrich) with NaOH (1 N). Moreover, the ferrous sulfate solution in 0.2 M hydrochloric acid and the 5 mM ferrozine solution were obtained using chemicals and reagents acquired from Sigma Aldrich (Steinheim, Germany). For the cytotoxicity assays, Dulbecco’s Modified Eagle’s Medium (DMEM), thiazolyl blue tetrazolium bromide (MTT), penicillin/streptomycin/neomycin mixture, as well as other reagents were acquired from Sigma-Aldrich, Germany.

### 4.2. Plant Material and Extract Preparation

*Tagetes erecta* (“Discovery Yellow” cultivar) was cultivated in environmentally friendly conditions in northeastern Romania in 2017. The flowers were harvested in September the same year, then dried in a well-ventilated space, away from light and deposited in the Pharmacognosy department of “Grigore T. Popa” University of Medicine and Pharmacy Iași. The plant material was then pulverized using a commercial blender. Ten grams of the powder was weighed and mixed with 200 mL methanol. The extraction was carried out at room temperature for 3 h, using a magnetic stirrer (DLAB MS-M-S10, Beijing, China). Afterward, the extract was filtered, and the organic solvent was evaporated using a rotary evaporator (100 mbar pressure, 45 °C temperature). The dry extract was kept at a temperature of 4 °C until different assays were carried out.

### 4.3. Isolation

The methanolic extract was resuspended in DMSO using ultrasonication and subjected to solid-phase extraction using a preconditioned RP-C_18_ column (100 × 80 mm i.d.; Cosmosil 140C_18_-PREP, 140 µm), followed by the exclusion of compounds with high polarity (using solutions of 1% MeOH *v*/*v* and 50% MeOH *v*/*v*, respectively). Subsequently, a polyphenolic-rich fraction was eluted with a solution containing 85% MeOH and 0.1% formic acid. This fraction was then further purified by elution with 100% MeOH using a Sephadex LH-20 column (970 × 34 mm i.d., Sigma-Aldrich, Steinheim, Germany). After performing this separation, four major fractions (Fr 1–4) were collected. The chemical composition of these fractions was monitored through LC-MS techniques.

### 4.4. LC-MS and Qualitative Analysis

The phytochemical analysis of the total methanolic extract was performed using high-resolution LC-MS analyses, with a Thermo Scientific Ultimate 3000RS chromatographic system [48]. The process was carried out on a Waters ACQUITY UPLC BEH C18 column (150 × 2.1 mm i.d.; 1.7 µm, Milford, MA, United States) at a temperature of 50 °C. The separation of constituents of interest was accomplished using a concave-shaped gradient (Dionex gradient curve 6) from 5% to 60% phase B for 25 min, with a flow rate of 0.55 mL/min. The mobile phase was represented by 0.1% (*v*/*v*) formic acid in distilled water (phase A) and 0.1% (*v*/*v*) formic acid in acetonitrile (phase B).

The photodiode array detector recorded absorbances in the 200–600 nm wavelength range, with 5 nm bandwidth and 10 Hz acquisition frequency. A flow splitter was used to divert the column effluent in a proportion of 1:3 between Q-TOF MS (Bruker Impact II HD, Bruker, Billerica, MA, United States) and a charged aerosol detector (CAD; Thermo Corona Veo RS) linked in parallel. The acquisition frequency for the CAD was 10 Hz.

The MS analyses were operated in both positive and negative ion mode, using electrospray ionization. Linear spectra were obtained in the 50 to 2000 *m/z* mass range, with 5 Hz acquisition frequency and the following parameters of the mass spectrometer: negative ion capillary voltage = 3.0 kV; positive ion capillary voltage = 4.5 kV; dry gas flow = 6 L/min; dry gas temperature = 200 °C; collision cell transfer time = 90 μs; nebulizer pressure = 0.7 bar. The obtained data were calibrated internally, with sodium formate introduced into the ion source via a 20 µL loop at the start of each separation. The chromatographic data was acquired and processed using Bruker DataAnalysis 4.3 software.

The four fractions that were collected after separation on the LH-20 column were investigated, taking into consideration their major constituents, using a Waters ACQUITY UPLC system (Waters Corp., Milford, MA, United States) equipped with a binary pump and MS and DAD detectors. The separation was carried out on a ACQUITY UPLC BEH C18 column (100 mm × 2.1 mm, 1.8 μm; Waters Corp., Milford, MA, United States) at 50 °C, with a flow rate of 500 μL/min. The mobile phase was represented by formic acid 0.1% (*v*/*v*) in purified water (solvent A) and formic acid 0.1% (*v*/*v*) in acetonitrile (solvent B). The separation was accomplished using a 7–80% solvent B gradient for 15 min.

The MS analyses were operated through electrospray ionization in both positive and negative modes. Measurements in the positive mode were correlated with UV–Vis detection in the 190–480 nm wavelength range. Linear spectra were obtained in the 100–2000 *m/z* mass range, with the following parameters of the mass spectrometer: positive ion capillary voltage = 3.1 kV; negative ion capillary voltage = 2.8 kV. The chromatographic data were acquired and processed using Waters MassLynx v.4.1 software.

### 4.5. NMR Spectroscopy

The 1D and 2D NMR spectra (^1^H, ^13^C DEPTQ, ^1^H–^13^C HSQC, ^1^H–^13^C HMBC, ^1^H–^13^C F2-coupled HSQC, ^1^H–^1^H COSY, 1D TOCSY) were acquired using an Avance III HD Ascend 500 MHz spectrometer (Bruker BioSpin, Rheinstetten, Germany), in DMSO-*d_6_* at 30 °C.

### 4.6. Antioxidant Activity Assays

#### 4.6.1. Lipoxygenase Inhibition

Lipoxygenase inhibition was assessed for the initial extract and Fr 4, according to the modified Malterud method [49]. A total of 50 µL lipoxidase from Glycine max in borate buffer (pH 9) was mixed with 50 µL of a sample/control solution of various concentrations in DMSO, and left to stand for 10 min. Afterward, 2 mL of 0.16 mM linoleic acid borate buffer was added, and the absorbance was measured for 90 s at 234 nm. The inhibition of lipoxygenase was calculated using the following formula: Inhibition (%) = (A_EFI_ − A_ECI_) × 100/A_EFI_; A_EFI_ is the difference between the absorbances of the enzyme solution without inhibitor at 90 and 30 s, respectively, while A_ECI_ is the difference between the enzyme-inhibitor solution absorbances at 90 and 30 s, respectively. Quercetin was used as positive control, and the IC_50_ values were expressed as µg/mL of the final solution. All experiments were performed in triplicate.

#### 4.6.2. Metal Chelation

The capacity of chelating ferrous ions was evaluated according to the method of Venditti et al., with some modifications [50,51]. Ferrous ions react with ferrozine, forming a pink complex with maximum absorbance at 562 nm. The existence of a chelating agent in the reaction medium will therefore lead to the decrease of the measured absorbance. A total of 0.2 mL of the sample solution, 0.74 mL of 0.1 M acetate buffer (pH 5.25), and 0.02 mL of 2 mM ferrous sulphate solution in 0.2 M hydrochloric acid were mixed for 10–15 s. Afterward, 0.04 mL ferrozine solution 5 mM was added, and the absorbance of the solution was measured after keeping it for 10 min in the absence of light, against a blank prepared under similar conditions. The metal chelating capacity was determined for the initial extract, and the investigated fraction using the following formula: Activity (%) = 100 × (Ac − As)/(Ac), where Ac is the absorbance of the control solution and As is the absorbance of the sample solution. Quercetin was used as positive control, and the EC_50_ values were expressed as mg sample/mL final solution. The experiments were carried out in triplicate.

### 4.7. Cytotoxicity Testing

The MTT assay is a popular colorimetric assay developed for cell viability screening. This test measures the reduction of yellow MTT to a purple formazan product carried out by mitochondrial dehydrogenases, and its absorbance is then measured spectrophotometrically. Dead cells lose their capacity of converting MTT into formazan. Consequently, color development can serve as an indicator of viable cells [52,53]. The cytotoxicity assessment was performed by the evaluation of cell viability in the presence of the investigated samples, using a direct contact MTT assay, which was carried out according to the protocols in the existing literature, as well as according to the recommendations of ISO 10993-5 [54,55]. The absorbance of the obtained solutions was measured at 570 nm, using isopropanol as blank.

Albino rabbit dermal fibroblasts, passage 5, were used for cytotoxicity tests. These cells had been isolated using the explant culture method, to obtain a homogenous population of cells [56].

The cytocompatibility of the samples was evaluated using 96-well plates, each well including a population of 5 × 10^3^ cells, in DMEM with 10% fetal bovine serum, 1% penicillin/streptomycin/neomycin mixture, and HAM/F12. The plates were eventually incubated at 37 °C, 5% CO_2_ atmosphere, and 97% humidity. The cells were treated with solutions of different concentrations (0.25 mg/mL, 0.5 mg/mL and 1 mg/mL) obtained from the initial extract and the investigated fraction, for 24, 48, and 72 h, respectively. The samples were dissolved in the culture medium with a content of 1% DMSO.

Cell viability was calculated using the following formula: Cell viability (%) = *As*/*Ac* × 100, where *As* is the absorbance of the sample culture and *Ac* is the absorbance of the control culture, which was not exposed to the investigated samples. All experiments were carried out in triplicate.

Cell viability studies were carried out in triplicate (*n* = 3) for each experiment and analyzed by means of two-way ANOVA. A *p*-value of less than 0.01 was accepted as significant.

## 5. Conclusions

The present study focused on a phytochemical, HR LC-MS analysis of a methanolic extract obtained from *T. erecta* flowers, which revealed the presence of over 50 compounds pertaining to different classes of plant metabolites, such as polyphenolic acids, flavonoids, amino acids, and tannins. Further purifications of the extract led to the obtaining of four fractions, the least complex fraction represented by a single flavonoid being characterized using NMR techniques. Therefore, this fraction containing over 85% quercetagitrin was further examined from a biological perspective, compared to the initial extract. The antioxidant and cytotoxicity assays generally revealed more promising results for the investigated fraction than for the total extract, which justifies further isolation of compounds from *T. erecta* to obtain compounds with more promising biologic actions and lower cytotoxicity, that could present important applications in the treatment of several conditions related to oxidative stress and inflammation.

## Figures and Tables

**Table 1:** Compounds identified in the *T. erecta* L. total methanolic extract using UHPLC–QTOF–MS/MS.

No.	Compound Name	RT * (min)	Formula	Error (ppm) **	mσ ***	Observed [M-H]^-^	Major Fragments (%)	Reference
1.	Quinic acid	0.63	C_7_H_12_O_6_	0.1	1.2	191.0561	-	[12]
2.	Quinic acid hexoside	0.63	C_13_H_22_O_11_	0.5	9.4	353.1088	173.0455 (100), 191.0560 (5)	-
3.	Dihexoside	0.63	C_12_H_22_O_11_	0.5	7.0	341.1088	-	-
4.	Galloyl hexoside	0.76	C_13_H_16_O_10_	0.5	16.0	331.0669	169.0137 (100), 211.0247 (85), 271.0457 (35), 125.0245 (19), 241.0356 (7)	[13]
5.	Shikimic acid hexoside	0.76	C_13_H_20_O_10_	0.4	15.3	335.0983	215.0522 (68)	-
6.	Theogallin	0.79	C_14_H_16_O_10_	0.3	9.6	343.0670	191.0560 (100)	[14]
7.	Phenylalanine	1.25	C_9_H_11_NO_2_	3.6	6.3	164.0711	-	[15]
8.	Syringic acid-hexoside I	1.76	C_15_H_20_O_10_	0.4	8.4	359.0982	197.0457 (100), 182.0067 (91), 138.0308 (48), 153.0546 (27)	[16]
9.	Methyl-gallic acid	1.81	C_8_H_8_O_5_	−0.8	45.6	183.0300	168.0062 (100), 124.0157 (97)	-
10.	Digalloyl-hexoside I	1.81	C_20_H_20_O_14_	−0.3	79.7	483.0782	271.0457 (100), 211.0245 (87), 169.0128 (75), 313.0572 (15), 423.0574 (10), 124.0191 (9)	[17]
11.	Tryptophan	1.94	C_11_H_12_N_2_O_2_	0.3	8.4	203.0825	116.0493 (100), 186.0526 (16)	[15]
12.	Digalloyl-hexoside II	2.20	C_20_H_20_O_14_	−0.3	15.0	483.0782	271.0457 (100), 211.0249 (93), 169.0139 (79), 347.0620 (36), 423.0571 (23), 313.0570 (17)	[18]
13.	Syringic acid-hexoside II	2.34	C_15_H_20_O_10_	−0.7	17.3	359.0986	197.0455 (100), 182.0221 (91), 138.0308 (48), 153.0546 (27)	[19]
14.	Digalloyl-dihexoside	2.44	C_26_H_30_O_19_	−0.4	26.0	645.1311	313.0569 (100), 271.0448 (82), 169.0139 (65), 493.1200 (57), 475.1103 (34)	-
15.	Digalloyl-hexoside III	2.60	C_20_H_20_O_14_	−0.6	1.5	483.0783	271.0460 (100), 211.0249 (51), 169.0140 (43), 313.0567 (27), 125.0237 (8)	[18]
16.	Galloyl-syringic acid-hexoside I	2.62	C_22_H_24_O_14_	0.1	21.5	511.1093	169.0142 (100), 125.0238 (31), 313.0557 (13), 197.0451 (13), 182.0246 (4), 271.0461 (4)	-
17.	Chlorogenic acid (5-CQA)	2.74	C_16_H_18_O_9_	−0.3	7.6	353.0879	191.0562 (100)	[20]
18.	Galloyl-syringic acid-hexoside II	3.11	C_22_H_24_O_14_	0.0	6.4	511.1093	169.0140 (100), 125.0237 (29), 197.0455 (21), 211.0245 (15), 271.0464 (13), 313.0566 (12)	-
19.	Trigalloyl-dihexoside	3.15	C_33_H_34_O_23_	2.2	21.4	797.1401	169.0137 (100), 313.0564 (49), 493.1206 (40), 475.1091 (32), 271.0461 (24)	[21]
20.	Syringic acid	3.57	C_9_H_10_O_5_	−1.5	4.4	197.0459	182.0221 (100), 166.9983 (37), 123.0071 (11)	[19]
21.	Trigalloyl-hexoside I	3.57	C_27_H_24_O_8_	−1.1	22.0	635.0897	169.0147 (100), 271.0460 (32), 211.0247 (26), 313.0572 (25), 125.0242 (18), 423.0579 (12)	[22]
22.	Galloyl-syringic acid-hexoside III	3.65	C_22_H_24_O_14_	−1.1	20.6	511.1099	169.0142(100), 197.0459 (98), 183.0298 (41), 125.0237 (37), 313.0542 (20)	[23]
23.	Syringic acid-(dihydroxydimethoxybenzoic acid)-hexoside I	3.83	C_24_H_28_O_15_	0.4	3.6	555.1353	197.0457 (100), 182.0224 (31), 153.0549 (23), 121.0283 (14), 138.0308 (7), 166.9987 (6)	[19]
24.	Sinapoyl alcohol	4.00	C_11_H_14_O_4_	0.4	10.0	209.0818	149.0585 (100)	[24]
25.	Syringic acid-(dihydroxydimethoxybenzoic acid)-hexoside II	4.19	C_24_H_28_O_15_	−0.1	0.9	555.1356	197.0458 (100), 153.0548 (30), 182.0222 (29), 121.0286 (17), 138.0308 (9), 166.9985 (4)	[19]
26.	Ellagic acid-hexoside I	4.41	C_20_H_16_O_13_	−1.1	3.6	463.0523	300.9993 (100), 271.9965 (3)	[25]
27.	Quercetagetin−7-*O*-(Ac-Pen-Hex)-3-*O*-hexoside I	4.59	C_34_H_40_O_23_	0.3	15.0	815.1885	317.0306 (100)	[19]
28.	Galloyl-eudesmic acid-hexoside I	4.62	C_23_H_26_O_14_	−0.3	6.9	525.1251	183.0304 (100), 168.0064 (27), 197.0459 (17), 139.0391 (15), 312.0492 (10), 124.0161 (10)	[19]
29.	Ellagic acid-hexoside II	4.68	C_20_H_16_O_13_	−1.2	75.4	463.0524	299.9915 (100)	[25]
30.	*N*-Malonylphenylalanine	4.97	C_12_H_13_NO_5_	−1.5	10.1	250.0725	164.0716 (100), 206.0814 (47), 147.0443 (21)	[26]
31.	Quercetagetin-3-*O*-hexoside	4.97	C_21_H_20_O_13_	−1.3	18.1	479.0837	316.0236 (100), 287.0210 (20), 271.0255 (11), 165.9909 (7), 243.0323 (4)	[19]
32.	Galloyl-eudesmic acid-hexoside II	5.02	C_23_H_26_O_14_	−0.9	4.1	525.1254	183.0303 (100), 168.0061 (34), 197.0459 (31), 225.0406 (15), 139.0387 (13), 124.0162 (10)	[19]
33.	Trigalloyl-hexoside II	5.26	C_27_H_24_O_8_	−0.7	27.5	635.0895	313.0577 (100), 169.0142 (91), 271.0461 (31), 295.0466 (19), 465.0641 (18), 211.0266 (18)	[22]
34.	Quercetagitrin (Quercetagetin-7-*O*-β-glucopyranoside)	5.33	C_21_H_20_O_13_	−0.9	1.5	479.0836	317.0304 (100)	[19]
35.	Syringic acid-(dihydroxydimethoxybenzoic acid)-hexoside III	5.68	C_24_H_28_O_15_	−1.1	25.2	555.1361	197.0452 (100), 313.0927 (64), 182.0242 (45), 153.0549 (40), 138.0315 (22), 223.0616 (20)	-
36.	Quercetagetin-7-*O*-dihexoside	5.69	C_27_H_30_O_18_	−0.5	6.8	641.1363	317.0303 (100)	[19]
37.	Quercetagetin-7-*O*-(Ac-Pen-Hex)-3-*O*-hexoside II	5.71	C_34_H_40_O_23_	0.0	36.5	815.1887	317.0309 (100)	[19]
38.	Quercetagetin-7-*O*-(galloyl-hexoside)	5.96	C_28_H_24_O_17_	0.4	8.9	631.0938	317.0307 (100)	[19]
39.	Ellagic acid	5.96	C_14_H_6_O_8_	−2.2	12.2	300.9997	300.9996 (100), 283.9964 (5), 229.0145 (5), 257.0097 (3), 245.0099 (3)	[25]
40.	Quercetagetin-3-*O*-hexoside-7-*O*-(galloyl-hexoside)	6.03	C_34_H_34_O_22_	−0.1	9.1	793.1470	317.0309 (100), 479.0829 (3), 631.0950 (1)	[19]
41.	Di-syringic acid hexoside I	6.41	C_24_H_28_O_14_	−0.7	3.4	539.1411	197.0460 (100), 182.0227 (71), 153.0549 (17), 166.9988 (12), 326.0645 (11), 239.0565 (9)	[19]
42.	Di-syringic acid hexoside II	6.74	C_24_H_28_O_14_	−1.9	11.7	539.1411	197.0458 (100), 182.0226 (77), 326.0644 (17), 153.0550 (14), 166.9987 (13), 239.0565 (11)	[19]
43.	Quercetin 3-*O*-hexoside	6.83	C_21_H_20_O_12_	−1.4	0.4	463.0888	301.0359 (100)	[19]
44.	Syringic acid-(hydroxytrimethoxybenzoic acid)-hexoside	7.01	C_25_H_30_O_15_	−1.6	5.0	569.1521	197.0458 (100), 182.0226 (30), 153.0550 (19), 269.0672 (14), 254.0438 (12), 121.0290 (10)	-
45.	Kaempferol 3-*O*-hexoside I	7.26	C_21_H_20_O_11_	−2.5	10.3	447.0944	285.0404 (100)	[27]
46.	Quercetagetin	7.41	C_15_H_10_O_8_	−2.6	4.2	317.0311	317.0310 (100), 271.0258 (10), 166.9991 (7), 243.0311 (5), 261.0410 (5), 299.0205 (5)	[28]
47.	Patulitrin	7.58	C_22_H_22_O_13_	−1.5	4.9	493.0995	315.0169 (100), 330.0406 (58), 287.0211 (49), 271.0253 (13)	[19]
48.	3,5 or 4,5 di-CQA	8.08	C_25_H_24_O_12_	−2.5	10.7	515.1208	191.0566 (100), 179.0352 (9), 173.0463 (2), 161.0241 (2)	[20]
49.	Kaempferol 3-*O*-hexoside II	8.40	C_21_H_20_O_11_	−4.2	23.2	447.0951	285.0408 (100), 257.0460 (35), 151.0029 (9)	[29]
50.	Isorhamnetin 3-*O*-hexoside	9.12	C_22_H_22_O_12_	−1.4	5.7	477.1045	299.0207 (100), 314.0444 (40), 271.0263 (30)	[19]
51.	8-Hydroxyquercetagetin	9.43	C_15_H_10_O_9_	−3.6	8.7	333.0264	165.9915 (100), 193.9862 (52), 243.0306 (49), 271.0254 (34), 287.0205 (31), 259.0250 (13)	[19]
52.	Quercetin	9.43	C_15_H_10_O_7_	−3.2	6.4	301.0363	301.0363 (100), 245.0465 (7), 255.0309 (5), 283.0270 (2)	[30]
53.	2Hβ,3-dihydro-euparin-14-*O*-β-D-glucoside	9.63	C_19_H_24_O_9_	−2.7	20.6	395.1358	233.0826 (100), 215.0720 (34), 217.0875 (25), 205.0880 (21), 175.0414 (9)	[31]
54.	Kaempferol	10.91	C_15_H_10_O_6_	−0.7	4.1	285.0407	-	[19]
55.	Patuletin	11.32	C_16_H_12_O_8_	−0.8	5.7	331.0462	316.0228 (100), 165.9912 (14), 287.0214 (8), 271.0258 (7)	[19]
56.	Isorhamnetin	13.59	C_16_H_12_O_7_	1.0	6.7	315.0507	271.0247 (100), 300.0277 (96), 243.0298 (70), 255.0299 (21), 165.9907 (12)	[19]
57.	Axillarin	14.38	C_17_H_14_O_8_	1.7	2.2	345.0610	287.0197 (100), 302.0428 (32), 259.0245 (25), 330.0383 (24)	[19]

* Retention time (RT). ** Mass accuracy measurements expressed in parts per million (ppm). *** Isotopic pattern fit factor (mσ).

**Table 2:** ^1^H- and ^13^C-NMR data of quercetagitrin (compound **34**; 500 and 125 MHz, DMSO-*d_6_*, 30 °C).

Position	δ_H_ (*J* in Hz)	δ_C_, Type
2		147.5, C
3		135.6, C
4		176.1, C
5		145.3, C
6		129.6, C
7		151.5, C
8	6.92 s	93.6, CH
9		148.1, C
10		105.1, C
1′		122.0, C
2′	7.70 d (2.2)	115.4, CH
3′		145.0, C
4′		147.8, C
5′	6.89 d (8.5)	115.5, CH
6′	7.53 dd (8.5, 2.2)	119.9, CH
3-OH	9.30 s	
5-OH	12.21 s	
6-OH	8.39 s	
3′-OH	9.24 s	
4′-OH	8.59 s	
β-Glcp		
1	5.01 d (7.3)	101.0, CH
2	3.37 dd (9.2, 7.3)	73.2, CH
3	3.33 t (8.7)	75.8, CH
4	3.21 t (8.7)	69.7, CH
5	3.47 ddd (7.7, 6.2, 1.7)	77.3, CH
6a	3.75 dd (11.0, 1.7)	60.6, CH_2_
6b	3.50 dd (11.0, 6.2)	
2-OH	5.38 brd (2.9)	
3-OH	5.09 brs	
4-OH	5.06 d (5.1)	
6-OH	4.64 t (4.6)	

**Table 3:** Antioxidant activity of *T. erecta* total extract and fraction 4 (Fr 4).

	Lipoxygenase Inhibition	Iron-Chelating Activity
Sample	IC_50_ (μg/mL Final Solution)	EC_50_ (mg/mL Final Solution)
Total extract	25.85 ± 0.67 *	0.390 ± 0.001
Fr 4	16.49 ± 0.19	0.529 ± 0.001
Quercetin	17.45 ± 0.33 *	0.417 ± 0.011

* Data already published [35].

## Data Availability

The data presented in this study are available in Supplementary Materials.

## Supplementary Materials

The following are available online, Figure S1. ^1^H NMR spectrum of compound **34** (500 MHz, DMSO*-d_6_*, 30 °C), Figure S2. ^13^C NMR spectrum of compound **34** (125 MHz, DMSO-*d_6_*, 30 °C).
