# Supplementary material for: Phytochemical Profile, Antioxidant Activity, and Cytotoxicity Assessment of Tagetes erecta L. Flowers
Source: Molecules. 2021 Feb 24;26(5):1201. doi: 10.3390/molecules26051201 (PMC7956293; doi:10.3390/molecules26051201)
Supplement: Supplementary file 1 [file molecules-26-01201-s001.pdf]

Supplementary Materials

Phytochemical Profile, Antioxidant Activity, and Cytotoxicity Assessment of *Tagetes erecta* L. Flowers

Ana Flavia Burlec [1,†], Łukasz Pecio [2,†], Solomiia Kozachok [2], Cornelia Mircea [3,*], Andreia Corciovă [1,*], Liliana Vereștiuc [4], Oana Cioancă [5], Wiesław Oleszek [2] and Monica Hăncianu [5]

1 Department of Drug Analysis, Faculty of Pharmacy, "Grigore T. Popa" University of Medicine and Pharmacy, 16 University Street, 700115 Iasi, Romania; flavia_burlec@hotmail.com (A.F.B.); acorciova@yahoo.com (A.C.)
2 Department of Biochemistry and Crop Quality, Institute of Soil Science and Plant Cultivation—State Research Institute, Czartoryskich 8, 24-100 Puławy, Poland; lpecio@iung.pulawy.pl (Ł.P.); skozachok@iung.pulawy.pl (S.K.); wieslaw.oleszek@iung.pulawy.pl (W.O.)
3 Department of Pharmaceutical Biochemistry and Clinical Laboratory, Faculty of Pharmacy, "Grigore T. Popa" University of Medicine and Pharmacy, 16 University Street, 700115 Iasi, Romania; corneliamircea@yahoo.com
4 Department of Biomedical Sciences, Faculty of Medical Bioengineering, "Grigore T. Popa" University of Medicine and Pharmacy, 16 University Street, 700115 Iasi, Romania; liliana.verestiuc@umfiasi.ro
5 Department of Pharmacognosy, Faculty of Pharmacy, "Grigore T. Popa" University of Medicine and Pharmacy, 16 University Street, 700115 Iasi, Romania; oana.cioanca@gmail.com (O.C.), mhancianu@yahoo.com (M.H.)
* Correspondence: corneliamircea@yahoo.com (C.M.); acorciova@yahoo.com (A.C.)
† These authors contributed equally to this work.

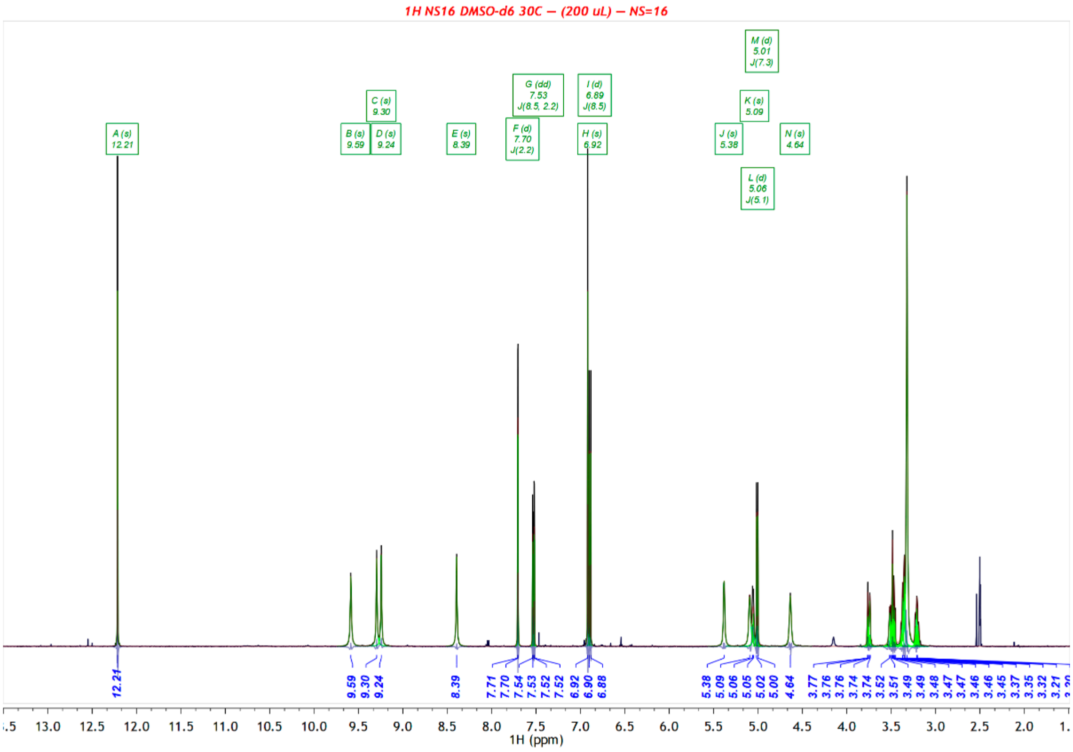

Figure S1. ^{1}H NMR spectrum of compound **34** (500 MHz, DMSO-d_6, 30 °C).

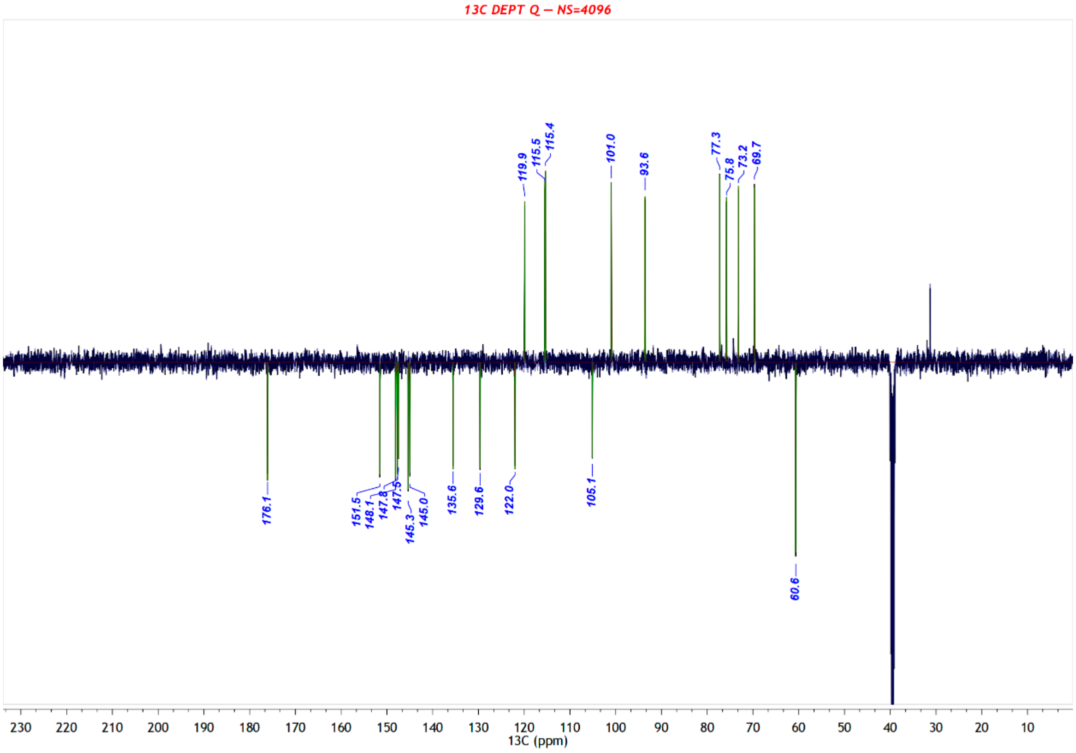

Figure S2. ^{13}C NMR spectrum of compound **34** (125 MHz, DMSO-d_6, 30 °C).
